# Sprouty2 and Spred1-2 Proteins Inhibit the Activation of the ERK Pathway Elicited by Cyclopentenone Prostanoids

**DOI:** 10.1371/journal.pone.0016787

**Published:** 2011-02-22

**Authors:** Carlota A. García-Domínguez, Natalia Martínez, Teresa Gragera, Andrea Pérez-Rodríguez, Diana Retana, Gonzalo León, Agustín Sánchez, José Luis Oliva, Dolores Pérez-Sala, José M. Rojas

**Affiliations:** 1 Unidad de Biología Celular, Área de Biología Celular y del Desarrollo, Centro Nacional de Microbiología, Instituto de Salud Carlos III (ISCIII), Majadahonda, Madrid, Spain; 2 Departamento de Biología Físico-Química, Centro de Investigaciones Biológicas, C.S.I.C., Ramiro de Maeztu 9, Madrid, Spain; Keio University, Japan

## Abstract

Sprouty and Spred proteins have been widely implicated in the negative regulation of the fibroblast growth factor receptor-extracellular regulated kinase (ERK) pathway. In considering the functional role of these proteins, we explored their effects on ERK activation induced by cyclopentenone prostanoids, which bind to and activate Ras proteins. We therefore found that ectopic overexpression in HeLa cells of human Sprouty2, or human Spred1 or 2, inhibits ERK1/2 and Elk-1 activation triggered by the cyclopentenone prostanoids PGA_1_ and 15d-PGJ_2_. Furthermore, we found that in HT cells that do not express Sprouty2 due to hypermethylation of its gene-promoter, PGA_1_-provoked ERK activation was more intense and sustained compared to other hematopoietic cell lines with unaltered Sprouty2 expression. Cyclopentenone prostanoids did not induce Sprouty2 tyrosine phosphorylation, in agreement with its incapability to activate tyrosine-kinase receptors. However, Sprouty2 Y55F, which acts as a defective mutant upon tyrosine-kinase receptor stimulation, did not inhibit cyclopentenone prostanoids-elicited ERK pathway activation. In addition, Sprouty2 did not affect the Ras-GTP levels promoted by cyclopentenone prostanoids. These results unveil both common and differential features in the activation of Ras-dependent pathways by cyclopentenone prostanoids and growth factors. Moreover, they provide the first evidence that Sprouty and Spred proteins are negative regulators of the ERK/Elk-1 pathway activation induced not only by growth-factors, but also by reactive lipidic mediators.

## Introduction

Sprouty was identified in *Drosophila melanogaster* as an antagonist of receptor tyrosine kinases (RTK) signaling during different morphogenetic processes, such as the development of the trachea, the eye, the wing and other tissues [1]–[5]. Currently, four mammalian genes have been identified that encode protein homologues for dSprouty [6]. The mammalian Sprouty isoforms have variable N-terminal sequences but share considerable cysteine-rich sequence homology in their C-termini. Ectopic overexpression of Sprouty2 inhibits fibroblast growth factor (FGF) and vascular endothelial growth factor (VEGF), but not epidermal growth factor- (EGF) induced ERK activation [7]. In addition, Sprouty2 Y55F mutant is unable to inhibit the ERK signaling after FGF stimulation [7]. Sprouty proteins have also been implicated in the negative feedback regulation of FGF signaling in embryogenesis [8], [9], angiogenesis [10] and myogenesis [11]. Although Sprouty2 binds Grb2 constitutively, through an interaction that involves the N-terminal SH3 domain of Grb2 and two Sprouty2 proline-rich stretches (residues 59–64 and 303–307) [12], [13], the inhibitory effect of Sprouty2 on FGF-induced ERK activation is independent of its Grb2-binding capacity [12] and it has no effect on Ras GTP loading [7], [12]. By contrast, it has also been suggested that Sprouty2 reduces Raf activation [7]. Sprouty2 localizes mainly in vesicular/endosomal and caveosome structures [2], [12], [14]–[17], although it has also been detected at the plasma membrane [12]. Indeed, Sprouty2 interferes with the progression from early to late endosomes affecting activated EGFR trafficking [17]. We have recently detected *hSprouty2* promoter hypermethylation in 37% of B-cell diffuse lymphoma cases, and found that this epigenetic alteration was associated with a significant decrease in the five-year survival rate [18].

Spred family members contain a C-terminal cysteine-rich Sprouty-related domain (SPR) [20], [21] with high homology to the C-terminal region of Sprouty proteins. Spred proteins also block RTK and cytokine receptors-triggered ERK activation [6], [19]. In addition, Sprouty inhibits PKC δ and Ca^+2^ signaling in *Xenopus*, whereas Spred proteins abolish the Ras-ERK signaling [22]. In mammals, Spred function is also focused on the Ras-ERK pathway [6], [19], where it blocks Raf activation [20].

Cyclopentenone prostanoids (cyP) are naturally occurring eicosanoids that show various biological activities, including antiviral [23] and antitumoral effects [24], modulation of the heat shock response [25], induction of oxidative stress [26], and apoptosis [27]. These prostanoids possess an α,β-unsaturated carbonyl group in the cyclopentene ring that favors the formation of Michael adducts with thiol groups in proteins, which is responsible for many of the biological effects of these compounds [28]–[31]. We have described that H-Ras, but not N- or K-Ras, is a target for the addition of the cyP 15-deoxy-Δ^12,14^-prostaglandin J_2_ (15d-PGJ_2_) since it forms a covalent bond with the cysteine 184 of H-Ras [32]. This effect is associated with the activation of the H-Ras-ERK pathway, increased proliferation of NIH-3T3 fibroblasts [32] and protection from apoptosis in MCA3D keratinocytes [33]. In accordance with these effects, we found that 15d-PGJ_2_ significantly enhanced the carcinogenic effect of DMBA/TPA in mice skin [33]. In addition, we also found that PGA_1_ binds to and activates H-, N- and K-Ras [34] mainly by binding to the cysteine 118 located in the GTP-binding motif, thus differing in the site of interaction of 15d-PGJ_2_ with H-Ras. Although the ERK pathway promotes some cellular responses induced by cyP [32], [33], [35], [36], we are not aware of any data suggesting that Sprouty proteins are involved in cyP signaling regulation.

This study was carried out to ascertain whether Sprouty and Spred proteins are able to inhibit ERK activation induced by stimuli different from agonists of tyrosine-kinase receptors, such as cyP.

## Materials and Methods

### Cell lines

HeLa cells [34] were maintained in DMEM (Invitrogen, Carlsbad, CA) supplemented with 10% fetal calf serum (FCS, Invitrogen), and the human hematological cell lines (HT and Karpas 422) [18] were grown RPMI 1640 (Invitrogen) containing 10% FCS.

### Transfections and antibodies

Transient transfections were performed using Jet-Pei™ (Polyplus-Transfection, Illkirch, France). All assays were done 48 h post-transfection. Monoclonal antibodies (mAb) to phospho-tyrosine (4G10) and Sprouty2 were from Upstate Biotechnology (Lake Placid, NY), and anti-phospho-ERK protein was from Cell Signaling (Beverly, MA). Rabbit polyclonal antibodies to ERK (ERK1/ERK2) and GST were purchased from Santa Cruz Biotechnology (Santa Cruz, CA), and anti-Spred1 and Spred2 antibodies were from Abcam (Cambridge, MA). Anti-HA and AU5 mAb were from Berkeley Antibody Company (Berkeley, CA), and anti-c-Cbl was from BD Transduction Laboratories (Franklin Lakes, NJ). Anti β-actin and β-laminin mAb, and recombinant human basic fibroblast growth factor (bFGF), and epidermal growth factor (EGF) were from Sigma-Aldrich (St. Louis, MO). PGA_1_ and 15d-PGJ_2_ were from Cayman Chemical (Ann Arbor, MI).

### DNA constructs

The plasmids pCEFL-KZ-HA, pCEFL-KZ-AU5, pCEFL-KZ-AU5-hSpry2 wt, pCEFL-KZ-AU5-hSpry2 Y55F, pCEFL-KZ-AU5-hSpry2 P59AP304A, pCEFL-KZ-HA-hSpry2 wt, pCEFL-HA-K-Ras 4B wt, and pCEFL-KZ-HA-ERK (1 and 2), have been previously described [11], [12], [37]. The cDNA of hSpred1 and hSpred2 were obtained by RT-PCR from mRNA of HeLa cells using the specific primers and providing sites Bam HI and Not I (hSpred1) or Bam HI and EcoRI (hSpred2) at the 5′ and 3′ ends, respectively. The amplified products were then subcloned into Bgl II and Not I/EcoRI sites within pCEFL-KZ-AU5. The sequences of the oligonucleotides utilized are available upon request.

### Ras-GTP detection

The plasmid pGEX-RBD, containing the Raf Ras-binding domain fused to glutathione S-transferase (GST) was kindly provided by D. Shalloway. The GST-fusion protein was purified following the previous described method [38] from *E. coli* Bl21 (DE3) harboring pGEX-RBD to express the fusion protein. In all Ras-GTP detection assays, transfected mammalian cells were lysed in cold lysis buffer [38], and 10 µg GST-RBD coupled to glutathione-sepharose beads, were added to the extracts and processed following the previous described method [38].

### Reporter gene analysis

HeLa cells were co-transfected with 0.6 µg of constructs encoding hSpry2 wt or mutants, 16 ng pCDNAIII-Gal4-Elk1, 0.1 µg pRL-TK (containing the *Renilla* luciferase gene under control of the HSV-TK promoter), and 0.3 µg pGal4-Luc (containing the *Photinus* luciferase gene controlled by six copies of a Gal4 responsive element). Assays were performed as previously described [37].

### Statistical analysis

Data were analyzed with SPSS software (Chicago). Results are expressed as the mean ± SD of the indicated number of experiments. Statistical significance was estimated with Student's *t* test for unpaired observations; *p*<0.05 was considered significant. For western blot analysis, we used linear correlations between increasing amounts of protein and its signal intensity.

## Results and Discussion

### Ectopic overexpression of Sprouty2, or Spred1-2, inhibits cyP-induced ERK/Elk1 pathway activation

**Figure 1:**
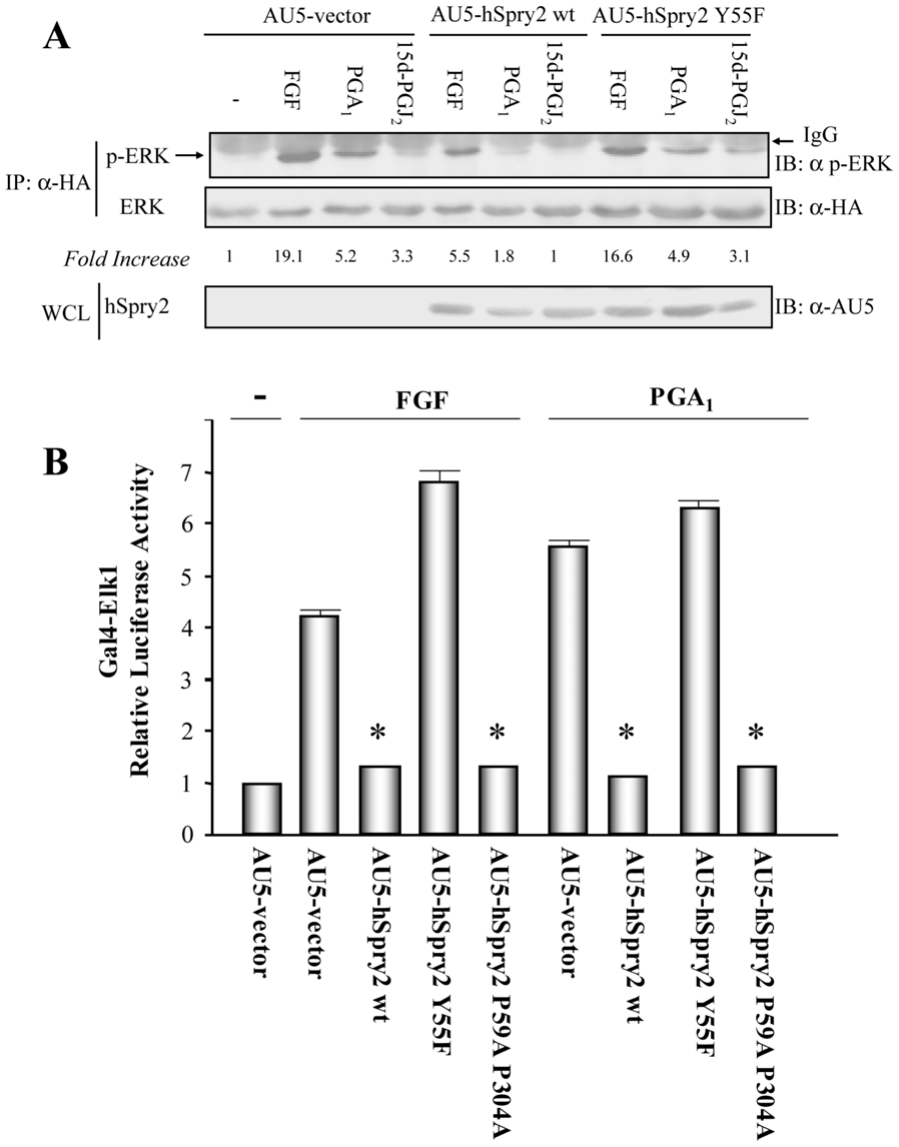
Overexpression of hSprouty2 (hSpry2) inhibits cyP-elicited ERK/Elk-1 pathway activation. (**A**) HeLa cells transiently co-transfected with pCEFL-KZ-HA-ERK1 and either pCEFL-KZ-AU5-hSpry2 wt, pCEFL-KZ-AU5-hSpry2 Y55F, or pCEFL-KZ-AU5 (AU5-vector), were serum-starved for 18 h and then incubated with vehicle (-), 50 ng/ml FGF, or 10 µM PGA_1_ (or 15d-PGJ_2_), for 15 min. Cell lysates were immunoprecipitated with anti-HA mAb, and analyzed by immunoblot using anti-p-ERK and -HA antibodies. Results were similar in three additional experiments. The factor by which values of p-ERK increased is estimated as mean of four separate assays (in each case with a SD lower than 10% of mean). The expression levels of AU5-hSpry2 constructs were detected by immunoblotting whole cell lysates (WCL) with anti-AU5 mAb (lower panel). (**B**) HeLa cells were co-transfected with pcDNAIII-Gal4-Elk-1, pGal4-Luc, and pRL-TK together with pCEFL-KZ-AU5 containing the indicated hSpry2 constructs in (A), and also pCEFL-KZ-AU5-hSpry2 P59A P304A. The transfected cells were serum-starved for 18 h, incubated with vehicle (-), either 50 ng/ml FGF, or 10 µM PGA_1_, for 8 h, and then assayed for luciferase activity. The data are the mean and SD of three separate assays performed in triplicate (* vs AU5-vector + FGF, or + PGA_1_: p<0.001).

Sprouty and Spred proteins have been implicated in the negative regulation of ERK signaling after stimulation by FGF [6], [19]. Here we examined whether human Sprouty2 (hSpry2) or human Spred (hSpred1 and hSpred2) can be negative regulators of ERK activation induced by cyP (PGA_1_ and 15d-PGJ_2_). For this purpose, we measured ERK phosphorylation promoted by these cyP in HeLa cells overexpressing HA-ERK2 and AU5-hSpry2 wt, or AU5-hSpry2 Y55F mutant (Fig. 1A). We found that, in a similar way to the described effects on FGF signaling, hSpry2 wt drastically reduced ERK activation after PGA_1_ or 15d-PGJ_2_ treatment. By contrast, hSpry2 Y55F mutant was unable to inhibit not only FGF-elicited ERK activation, as previously reported [6], but also that of cyP (Fig. 1A). Similar results were obtained when HA-ERK1 was used (not shown). We also tested Elk1 activation, a transcription factor downstream of ERK. hSpry2 wt, but not hSpry2 Y55F, blocked FGF- and PGA_1_-induced Elk1 activation (Fig. 1B), data which supports the results obtained by the analysis of phospho-ERK levels. In addition hSpry2 P59A P304A, a double mutant which does not bind Grb2 [12], also reduced Elk1 activation provoked by those stimuli, suggesting that the inhibitory mechanism of Sprouty2 on cyP-induced ERK/Elk1 activation is independent of Grb2-Sprouty2 interaction, as we previously demonstrated for FGF signaling [12].

**Figure 2:**
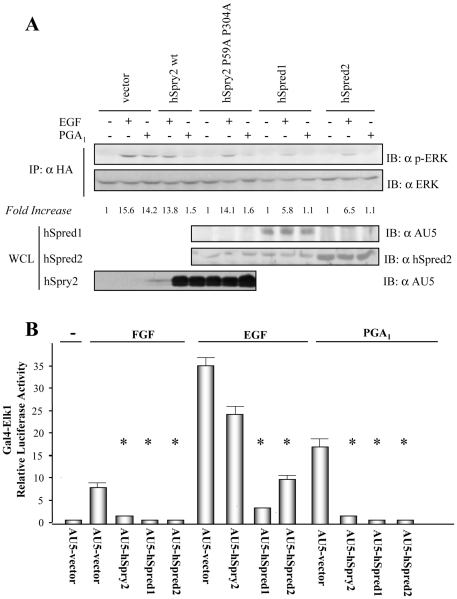
hSpred1 and hSpred2 block PGA_1_-induced ERK/Elk-1 pathway activation. (**A**) HeLa cells transiently co-transfected with pCEFL-KZ-HA-ERK1 and either pCEFL-KZ-AU5-hSpry2 wt, pCEFL-KZ-AU5-hSpry2 P59A P304A, pCEFL-KZ-AU5-hSpred1, pCEFL-KZ-AU5-hSpred2, or pCEFL-KZ-AU5 (AU5-vector), were serum-starved for 18 h and then incubated with vehicle (-), either 100 ng/ml EGF, or 10 µM PGA_1_, for 15 min. Cell lysates were immunoprecipitated with anti-HA mAb and analyzed by immunoblot using anti-p-ERK and -HA antibodies. Results were similar in three additional experiments. The factor by which values of p-ERK increased is estimated as mean of four separate assays (in each case with a SD lower than 10% of mean). The expression levels of AU5-hSpry2, AU5-hSpred1, or AU5-hSpred2, constructs were detected by immunoblotting WCL with the corresponding mAb (lower panels). (**B**) HeLa cells were co-transfected with the plasmids pcDNAIII-Gal4-Elk-1, pGal4-Luc, and pRL-TK together with pCEFL-KZ-AU5 containing the indicated hSpry2, or hSpred1, or hSpred2, constructs denoted in (A). The transfected cells were serum-starved for 18 h, incubated with vehicle (-), either 100 ng/ml EGF, or 50 ng/ml FGF, or 10 µM PGA_1_, for 8 h, and then assayed for luciferase activity. The data are the mean and SD of three separate assays performed in triplicate (* vs AU5-vector + FGF, or AU5-vector + EGF, or + PGA_1_: p<0.001).

We observed that, as it was expected [7], hSprouty2 wt or hSpry2 P59AP304A double mutant were unable to shut down the ERK/Elk1 signaling pathway after EGFR activation (Fig. 2), whereas hSpred1 or hSpred2 reduced phospho-ERK levels stimulated by EGF (Fig. 2A). We found that hSpred1 and hSpred2 also inhibited PGA_1_-triggered ERK activation (Fig. 2A), and both hSpred proteins abolished EGF, FGF, or PGA_1_-induced Elk1 activation (Fig. 2B).

All these data suggest that both Sprouty2 and Spred1/2 proteins negatively-regulate the cyP-dependent ERK/Elk1 pathway activation.

### Absence of Sprouty2 expression correlates with enhanced cyP-triggered ERK/Elk1 pathway activation

**Figure 3:**
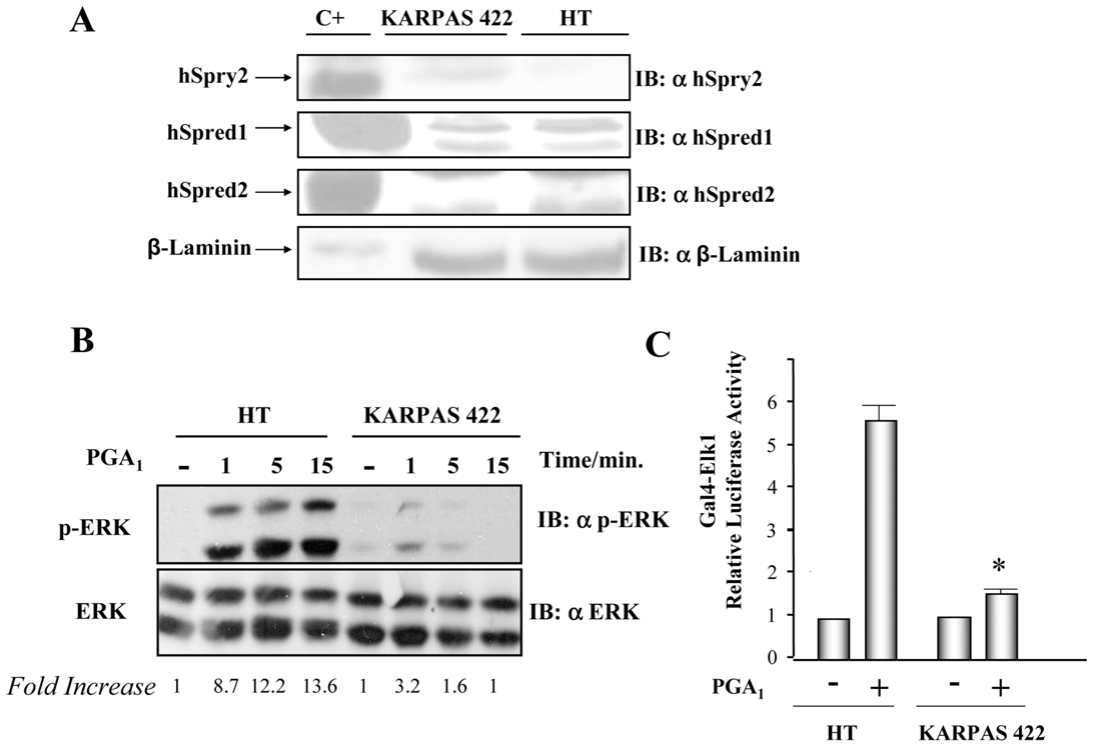
PGA_1_-induced ERK/Elk-1 pathway activation depends of hSpry2 expression. (**A**) Expression levels of hSprouty2 (hSpry2), hSpred1, and hSpred2 proteins in HT and Karpas 422 cell lines. Cells were lysed under appropriate conditions and equal amounts of proteins were analyzed by immunoblot. Expression levels were assessed using specific anti-hSpry2, -hSpred1, -hSpred2 and -β Laminin (as loading control) antibodies. C+ (positive control) corresponds to HeLa cells transiently co-transfected with pCEFL-KZ-AU5-hSpry2 wt, pCEFL-KZ-AU5-hSpred1, and pCEFL-KZ-AU5-hSpred2. The figure is from a representative experiment that was repeated two times more with similar results. (**B**) HT and Karpas 422 cell lines (5·10^6^ cells/point) were stimulated with 10 µM PGA_1_, for the indicated times. Cells were then lysed under appropriate conditions and equal amounts of proteins were analyzed by immunoblot. ERK phosphorylation levels (p42 and p44 proteins) were assessed using specific anti-phospho and total antibodies (as loading control). Results were similar in three additional experiments. The factor by which values of p-ERK increased is estimated as mean of four separate assays (in each case with a SD lower than 10% of mean). (**C**) HT and Karpas 422 cells were co-transfected with pcDNAIII-Gal4-Elk-1, pGal4-Luc, and pRL-TK. The transfected cells were serum-starved for 18 h, incubated with vehicle (-), or 10 µM PGA_1_, for 8 h, and then assayed for luciferase activity. The data are the mean and SD of four separate assays performed in triplicate (* HT + PGA_1_ vs Karpas 422 + PGA_1_: p<0.001).

We have previously demonstrated that human *Sprouty2* promoter gene is hypermethylated in the HT cell line (derived from a B-cell diffuse lymphoma) [18]. Expression analysis of *hSprouty2* by RT-PCR and WB indicated the absence of hSprouty2 (mRNA and protein) in this cell line [18]; in contrast, the Karpas 422 cell line (also derived from a B-cell diffuse lymphoma), but without epigenetic alterations in the *hSprouty2* promoter [18], showed hSprouty2 expression (Fig. 3A). In addition, we found that both cell lines express hSpred1 but not hSpred2 protein (Fig. 3A); curiously hSpred1 protein was detected as a double band, probably due to post-translational modifications [6], [19]. We therefore analyzed whether the absence of hSprouty2 in the HT cells affected ERK activation promoted by PGA_1_. We found that PGA_1_-elicited phospho-ERK levels in HT cells were higher than in Karpas 422 cells (Fig. 3B). These results concur with Elk1 functional activity because we observed higher activation of this transcription factor in HT cells versus Karpas 422 cells after PGA_1_ stimulation (Fig. 3C).

All these results are consistent with the expression of hSprouty2 in Karpas 422 cells, and its absence from HT cell line, and support the hSprouty2 role as negative regulator of cyP-triggered ERK pathway activation independently of hSpred1/2 expression levels.

### Overexpression of hSprouty2 does not reduce PGA_1_–induced K-Ras 4B activation

**Figure 4:**
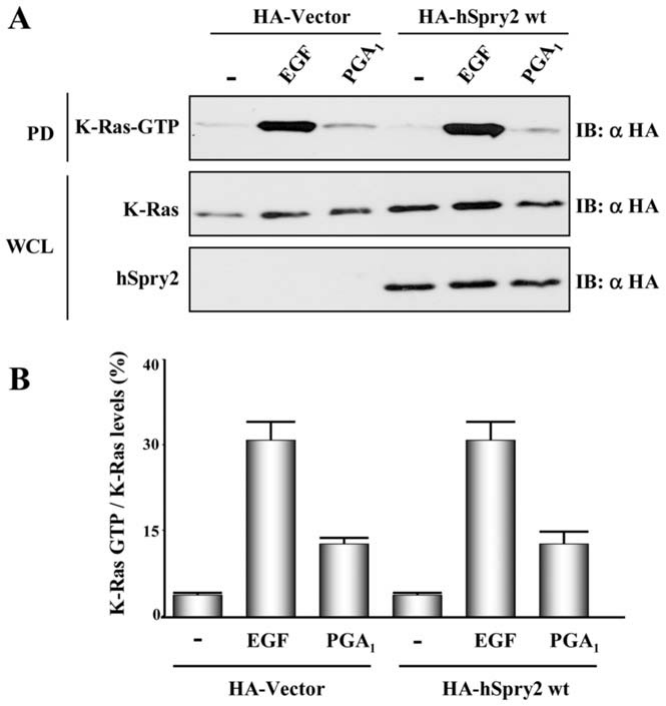
hSprouty2 does not affect PGA_1_-induced K-Ras 4B activation. (**A**) HeLa cells transiently co-transfected with pCEFL-KZ-HA-K-Ras 4B wt and pCEFL-KZ-HA-hSpry2 wt, or pCEFL-KZ-HA (vector), were serum-starved for 18 h and then incubated with vehicle (-), either 100 ng/ml EGF, or 10 µM PGA_1_, for 15 min. Ras-GTP was recovered from cell lysates by binding to immobilized GST containing the Ras·GTP binding domain of Raf (GST-RBD) and detected by immunoblotting with anti-HA mAb (upper panel). The expression levels of HA-K-Ras 4B wt and HA-hSpry2 wt were detected by immunoblotting WCL with the corresponding anti-HA mAb (lower panel). Results were similar in five independent assays. (**B**) Quantitative analysis of K-Ras-GTP standardized to K-Ras levels for the same type of experiments indicated in (A). The histogram shows the mean and SD of five separate assays.

Several studies have proposed that Sprouty proteins inhibit RTK-dependent Ras activation [2], [39], although we have not detected any effect of human Sprouty2 on K-Ras 4B activation levels promoted after RTK stimulation [12]. We previously showed that PGA_1_ binds to cysteine 118 of Ras, a residue located in the GTP-binding pocket, which correlates with Ras activation [34]. We thus investigated whether hSprouty2 was able to reduce the K-Ras 4B GTP-loading levels elicited by PGA_1_. Although treatment with PGA_1_ induced lower K-Ras 4B·GTP levels than EGF stimulation, in both cases hSprouty2 was unable to diminish K-Ras 4B activation (Fig. 4A–B). Our data therefore indicate that hSprouty2 does not have any effect on K-Ras 4B activation induced by EGF, FGF (not shown, and [12]) or PGA_1_ (Fig. 4A–B). These results support that in mammalian cells, unlike it happens in *Drosophila* [2], Sprouty2 inhibits ERK pathway downstream of Ras, probably by diminishing Raf activation [7], [10] independently of the type of stimulus (RTK or cyP).

### PGA_1_ treatment does not induce tyrosine phosphorylation of hSprouty2

**Figure 5:**
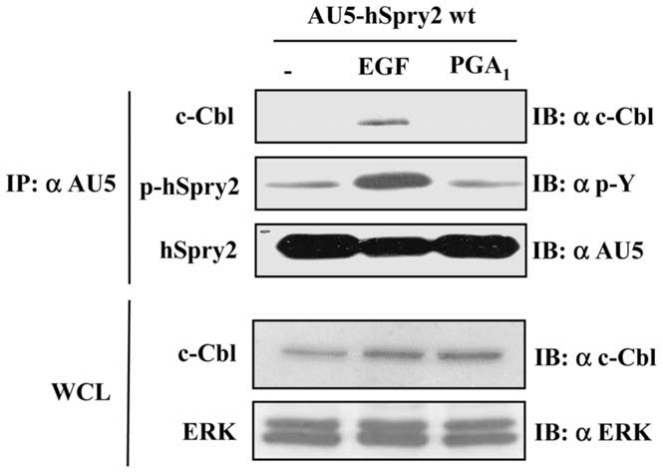
hSprouty2 is not tyrosine-phosphorylated by PGA_1_ treatment. HeLa cells transiently transfected with pCEFL-KZ-AU5-hSpry2 wt, were serum-starved for 18 h and then incubated with vehicle (-), 100 ng/ml EGF, or 10 µM PGA_1_, for 15 min. Cell lysates were immunoprecipitated with anti-AU5 mAb and analyzed by immunoblot using anti-p-Y, -c-Cbl, and -AU5 antibodies. Expression levels of endogenous c-Cbl were detected by immunoblotting with the appropriate anti-c-Cbl antibody (lower blot), and using anti-ERK as loading control. Results were similar in three additional experiments.

As it is shown above, hSpry2 Y55F mutant was unable to inhibit cyP-induced ERK/Elk1 pathway activation, whereas hSpry2 P59A P304A double mutant (and hSpry2 P59A single mutant, not shown) showed a potent inhibitory effect (Fig. 1 and 2B). Mutation of tyrosine 55 of Sprouty2 abrogates its inhibitory properties in FGF signaling [7], [11], [12]. In addition, both, Y55F and P59A mutations impair Sprouty2 phosphorylation at tyrosine 55 [40], [41] after RTK stimulation, thus preventing Sprouty2 interaction with the SH2 domain of c-Cbl [14], [15]. In order to confirm whether tyrosine phosphorylation/c-Cbl binding are dispensable events for the inhibitory effect of Sprouty2 on PGA_1_ signaling, we explored if cyP triggered this post-translational modification of Sprouty2. PGA_1_ was unable to induce hSprouty2 tyrosine-phosphorylation, or hSprouty2 binding to c-Cbl, in sharp contrast to the results obtained after EGF treatment (Fig. 5), and in agreement with a PGA_1_ signaling pathway independent of RTK activation. All these results suggest that other aspects relative to tyrosine 55 but unrelated to its phosphorylation and binding affinity to c-Cbl are modulating Sprouty2 functionality, and that Sprouty2 inhibition of PGA_1_-dependent ERK activation does not need of Sprouty2 interaction with c-Cbl. The detailed aspects of this mechanism will be the subject of future investigations.

Activation of the ERK-Elk pathway by cyP could occur at various levels. Evidence demonstrates a correlation between the covalent modification of Ras proteins by cyP and Ras-ERK pathway activation. However, other mechanism for Ras or ERK activation could be envisaged, including oxidative stress induction by cyP or their interaction with other cellular targets [42]. Besides their binding to PPARs, cyP have been reported to interact with membrane receptors, including G-protein coupled receptors, and ion channels. Several cyP of the J series have been reported to connect with the DP2 receptor, one of the receptors for PGD_2_, also known as CRTH2 (for chemotactic receptor of TH2 cells) [43]. The dissociation constants for various cyP are in the nanomolar range, with PGD2 being more potent than D12-PGJ_2_ and this PG more potent than 15d-PGJ_2_. It has also been reported the interaction of D12-PGJ_2_ and 15d-PGJ_2_ with DP1 [43]. Whereas DP1 is coupled to Gαs-type of G proteins and its activation triggers an increase in cAMP levels, DP2 is thought to be coupled to Gαi/o and its activation induces high intracellular Ca^2+^ levels; this could contribute to the modulation of Ras protein activity through RasGRP. CRTH2 activation of Gαi proteins has also been reported to stimulate PI3-K, MAPK and PLCγ [44], and both ERK, a member of MAPK family, and PLCγ can be inhibited by Sprouty2 and Spred1/2 proteins [6]. PGD_2_, as well as its metabolites dk-PGD_2_, PGJ_2_, D12-PGD_2_, D12-PGJ_2_, 15d-PGD_2_ and 15d-PGJ_2_, have been shown to be potent eosinophilia activators with respect to chemotaxis, actin polymerization, L-selectin shedding and CD11b upregulation, in a CRTH2-dependent manner [45], [46]. The role of DP2 in mediating the effects of 15d-PGJ2 appears nevertheless to be largely cell-dependent. DP2 receptor antagonists appear to block 15d-PGJ_2_-elicited enhancement of NGF-induced neurite outgrowth [47]. However, DP2 appears not to be involved in PGD_2_ or 15d-PGJ_2_ effects on inflammatory resolution [48], on the expression of TLR2 in brain glia [49] or in the derangement of vimentin cytoskeleton and the inhibition of iNOS induction by 15d-PGJ_2_ in mesangial cells [50]. Moreover, to the best of our knowledge there are no evidences of PGA_1_ interaction with these receptors. In addition, cyP have been recently found to interact with other G-protein coupled receptors. 15d-PGJ_2_ has been reported to activate kappa/delta opioid receptors, as suggested by pharmacological evidences [51]. Interestingly, several cyP have been reported to covalently bind and activate the ion channel TRPA1, leading to nociceptive responses [52]. Finally, results from our group have identified the G-protein b2-like1 subunit as a potential target for covalent modification by PGA_1_ [53]. However, the elucidation of the functional significance of these results requires further study.

In conclusion, all the results presented in this study support that the ERK/Elk-1 pathway activation induced by cyP (PGA_1_ and 15d-PGJ_2_) treatment can be negatively-modulated by Sprouty2 and Spred1-2 proteins. These data suggest that the functional role of these proteins may be relevant not only as regulators of RTK (or G-protein-coupled receptors) signaling [6], but also as modulators of the ERK pathway activation dependent on Ras·GTP loading triggered by other stimuli, such as cyP [32]–[34].

**Figure 6:**
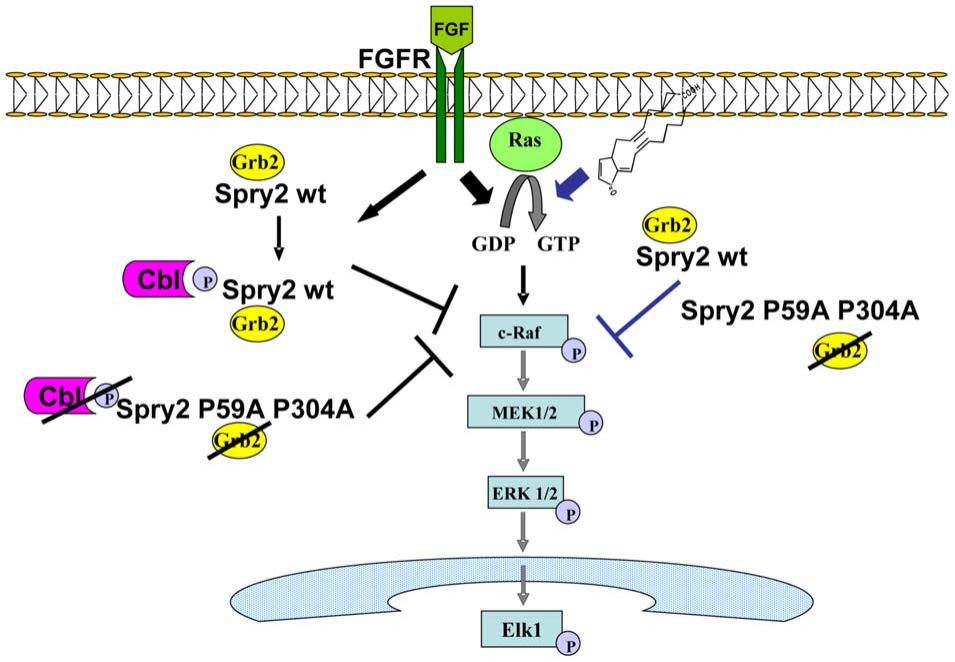
Model of action of hSprouty2 on cyP-triggered Ras/Raf/MEK/ERK/Elk1 signaling pathway. PGA_1_ binds to cysteine 118 of Ras and induces Ras·GTP loading and Raf/MEK/ERK/Elk1 pathway activation. Overexpression of Sprouty2 (Spry2) wt or Spry2 P59A P304A double mutant (unable to bind Grb2) block cyP and FGF-elicited ERK phosphorylation but not Ras activation, probably due to an effect on Raf signaling. However, Spry2 Y55F mutant does not inhibit cyP and FGF-induced ERK pathway activation, although treatment with PGA_1_ was unable to promote tyrosine-phosphorylation of Spry2; in sharp contrast, RTK activation (mainly EGFR) provokes tyrosine-phosphorylation of Spry2 (at Y55 residue) increasing its binding affinity to the SH2 domain of c-Cbl, which leads to Spry2 ubiquitination.

Our data indicate, that the molecular activity of Sprouty2 on cyP signaling would be downstream of Ras (probably at the level of c-Raf activation), without having any effect on Ras·GTP levels elicited by these prostanoids (Fig. 6). In addition, Sprouty2 P59A P304A double mutant, which lacks the capacity to bind to Grb2 [12], inhibits the ERK/Elk-1 pathway after PGA_1_ treatment to a similar extent as Sprouty2 wt, demonstrating that the Sprouty2 activity to repress the PGA_1_/ERK/Elk-1 pathway does not require Grb2 binding (Fig. 6). Finally, we found that although Sprouty2 Y55F mutant did not block cyP-induced ERK and Elk-1 activation, the treatment with PGA_1_ was unable to provoke *in vivo* tyrosine-phosphorylation of Sprouty2, and c-Cbl/Sprouty2 binding (Fig. 6); however, RTK stimulation (mainly EGFR) induces tyrosine-phosphorylation of Spry2 (at Y55 residue) enhancing its binding affinity to the SH2 domain of c-Cbl, which leads to Spry2 ubiquitination [6]. Our data, suggest that, independently of phosphorylation of tyrosine 55 and c-Cbl binding, this tyrosine residue might be involved in additional functions, unexplored to date, which can exert a pivotal role in the molecular inhibitory mechanism of Sprouty2 on the cyP signaling.
